# Inhibition of miR-141 and miR-200a Increase *DLC-1* and *ZEB2* Expression, Enhance Migration and Invasion in Metastatic Serous Ovarian Cancer

**DOI:** 10.3390/ijerph17082766

**Published:** 2020-04-17

**Authors:** Norhazlina Abdul Wahab, Zahreena Othman, Noor Wahidah Mohd Nasri, Mohd Helmy Mokhtar, Siti Fatimah Ibrahim, Adila A. Hamid, Raja Affendi Raja Ali, Norfilza Mohd Mokhtar

**Affiliations:** 1 Department of Physiology, Faculty of Medicine, Universiti Kebangsaan Malaysia, Kuala Lumpur 56000, Malaysia; hazlina@ukm.edu.my (N.A.W.); othmanzahreena@yahoo.com (Z.O.);; 2 Department of Medicine, Faculty of Medicine, Universiti Kebangsaan Malaysia, Kuala Lumpur 56000, Malaysia

**Keywords:** serous ovarian cancer, metastasis, microRNA, MIRN141 microRNA, MIRN200 microRNA

## Abstract

The role of microRNA (miRNA) in ovarian cancer has been extensively studied as a regulator for its targeted genes. However, its specific role in metastatic serous ovarian cancer (SOC) is yet to be explored. This paper aims to investigate the differentially expressed miRNAs in metastatic SOC compared to normal. Locked nucleic acid PCR was performed to profile miRNA expression in 11 snap frozen metastatic SOC and 13 normal ovarian tissues. Functional analysis and regulation of their targeted genes were assessed in vitro. Forty-eight miRNAs were significantly differentially expressed in metastatic SOC as compared to normal. MiR-19a is a novel miRNA to be upregulated in metastatic SOC compared to normal. *DLC1* is possibly regulated by miR-141 in SOC. MiR-141 inhibition led to significantly reduced cell viability. Cell migration and invasion were significantly increased following miRNA inhibition. This study showed the aberrantly expressed miRNAs in metastatic SOC and the roles of miRNAs in the regulation of their targeted genes and ovarian carcinogenesis.

## 1. Introduction

An increasing trend of ovarian cancer was observed in countries with socioeconomic growth, which could be attributed to changes in lifestyle [1]. Ovarian cancer is divided based on its histological subtypes, which are serous, mucinous, endometrioid and clear cell types [2,3]. Serous ovarian cancer (SOC) stands as the most common histological subtype, which accounts for 70% to 80% of all ovarian cancer deaths [4]. Malignant serous tumors contribute to almost half of all malignant ovarian tumors, which occur most frequently in the sixth decade of life.

At the time of diagnosis, most malignant serous tumors would have spread extensively, mainly to the peritoneum, which reflects on the poor survival rates of the disease. The five-year survival rate for patients with stage III and stage IV SOCs are only 25% and 9%, respectively [5]. Thus, the discovery of clinically useful biomarkers for diagnostic and therapeutic purposes is of utmost importance in overcoming the disease. A critical step towards achieving this goal is by establishing a unique repertoire of microRNA (miRNA) expression in metastatic SOC and normal ovarian tissues.

Over recent years, increasing evidence has revealed the roles of small non-coding single-stranded RNAs, called miRNAs, in ovarian cancer tumorigenesis and metastasis. Post-transcriptionally, miRNAs repress gene expression by recognizing complementary target sites in the 3′ untranslated region of targeted messenger RNA (mRNA). The expression of targeted mRNAs is silenced either by translational repression or by mRNA degradation. Each miRNA may target a number of different mRNAs and, similarly, a single mRNA can be targeted by several miRNAs [6,7]. Currently, it is well-known that aberrantly expressed miRNAs contribute to cancer development. High miRNA expression in a tumor may lead to oncogenesis via the downregulation of its targeted tumor suppressor, whereas low miRNA expression may cause oncogene activation [8]. MiRNAs involved in metastasis are denoted as metastamiRs, which play substantial roles in cell growth, epithelial-to-mesenchymal transition (EMT), cell adhesion, invasion and migration, apoptosis and angiogenesis [7]. In ovarian cancer, several reports have identified pro-metastatic and antimetastatic miRNAs. For instance, miR-205 has been shown to enhance motility [9], and miR-31 is believed to reduce cell migration and invasion in ovarian cancer cells [10].

Expression patterns of miRNAs correlate with ovarian cancer histological subtypes, tumor stage, chemoresistance, cancer recurrence and survival, making miRNAs valuable as tissue and blood-based biomarkers [11,12,13,14]. However, most of the miRNA profiling studies to date reported expression patterns across different ovarian cancer histological subtypes, specimen types (tumor tissues vs. cell lines), chemotherapy status (post-chemo vs. chemo naïve), heterogeneity of the tumor, tumor stage and grade, profiling platforms, RNA isolation methods and the type of normal controls used [9,10,15,16]. These differences contribute to the partial overlap of the differentially expressed miRNAs in ovarian cancer. In particular, very few profiling studies are looking specifically into the serous subtype of ovarian cancer with metastatic evidence. One study has successfully demonstrated how miRNA expression profiles differ in omental metastases of SOC compared to primary tumors and how these differences influence chemotherapy [17]. Another study identified exosomal miR-200b and miR-200c in the serum of patients with advanced epithelial ovarian cancer, suggesting the possible active role of exosomal secretion [18]. Our group has documented aberrantly expressed miRNAs in metastatic SOC compared to normal using formalin-fixed and paraffin-embedded tissue samples [11]. This study expands the body of current literature by examining miRNA expression profiles in metastatic SOC fresh tissue samples compared to normal and demonstrating how these miRNAs may influence ovarian cancer growth and progression.

## 2. Materials and Methods

### 2.1. Human Tissue Samples

Twenty-four unmatched snap frozen ovarian tissue samples were collected at the time of surgery at the Universiti Kebangsaan Malaysia Medical Centre (UKMMC) after obtaining informed consent from the patients. Ethics approval was obtained from the UKM Research Ethics Committee (Ref: UKM 1.5.3.5/244/UKM-GUP-2011-286). The tissue samples consisted of 11 SOC (stages III and IV) and 13 unmatched normal ovarian tissues. Cancer tissues were taken from primary sites with metastatic evidence in the peritoneal cavity, and normal tissues were taken from patients undergoing total abdominal hysterectomy with bilateral salphingo-oophorectomy for benign gynecological conditions. The consultant pathologist verified the cancer samples to be serous adenocarcinoma, with more than 80% of cancer cells present based on hematoxylin and eosin staining. The normal samples comprised of normal ovarian epithelial tissues and were confirmed to be free of any pathology, including benign cysts. All tissue samples were chemotherapy-naïve. The patient information of the ovarian tissue samples used in the study is shown in Table 1.

### 2.2. MicroRNA Expression Profiling and Validation

Total RNA was purified using QIAzol lysis reagent and miRNeasy Mini Kit (Qiagen, Valencia, CA, USA; Cat No./ID: 217004) according to the manufacturer’s instructions. RNA quantity and quality were determined using the RNA 6000 Nano Kit and Bioanalyzer 2100 (Agilent Technologies, CA, USA). RNA template, at a concentration of 5 ng/µL, was reversed-transcribed to cDNA using Universal cDNA Synthesis Kit (Exiqon, Vedbaek, Denmark; Cat No: 203300). Differentially expressed miRNAs in cancer compared to normal was determined using miRCURY LNA Universal RT Cancer Focus microRNA PCR Panel, 96-well (V1.AF) and ExiLENT SYBR^®^ Green Master Mix (Exiqon, Vedbaek, Denmark; Product No: 203820, 203823) according to the manufacturer’s protocols. Eight upregulated miRNAs, namely miR-106a, miR-18a, miR-203, miR-93, miR-141, miR-7, miR-20a and miR-200a, were validated using the miRCURY LNA Universal RT Pick & Mix PCR Panels (Exiqon, Vedbaek, Denmark; Product No: 203801).

### 2.3. Statistical Analysis for microRNA Expression Profiling

The C_T_ values obtained from qPCR results were imported into the Exiqon GenEx Version 5.4.2 software (Exiqon, Vedbaek, Denmark) in .txt format. Data preprocessing was performed, and the reference gene was selected using NormFinder. MiRNA expression profiling was performed using the Kruskal-Wallis and LIMMA statistical tests with *p* < 0.05 and fold-change between <−2 and >2. The data points that fell beyond the upper quartile + 1.5 inter-quartile distance or the lower quartile −1.5. inter-quartile distance in the box plot were considered outliers, and they were replaced with the group median. The lower and upper quartiles were defined by the 25th and 75th percentiles, respectively. The R programming software was then employed to generate the heatmap diagram and hierarchical clustering of the differentially expressed miRNAs in metastatic SOC compared to normal tissues.

### 2.4. Bioinformatics Analysis of microRNA Targeted Genes

The Oncomine database (http://www.oncomine.org) was employed to determine candidate genes involved in metastatic SOC. Predicted targeted genes for miR-141 and miR-200a were identified using TargetScan (http://www.targetscan.org), mirWalk (http://www.umm.uniheidelberg.de/apps/zmf/mirwalk/mirnatargetpub.html) and miRDB (http://mirdb.org/miRDB). Gene lists from the Oncomine dataset and miRNA target prediction tools were overlapped using a Genn-Venn diagram to determine the overlapping candidate genes in all databases.

### 2.5. Cell Culture and Transfection

The ovarian cancer cell lines Caov3 (ovarian adenocarcinoma) and SKOV3 (ovarian adenocarcinoma, ascites) were obtained from the American Type Culture Collection (ATCC, Rockville, MD, USA) and cultured in DMEM and McCoy’s 5A, respectively, supplemented with 10% fetal bovine serum and 1% penicillin-streptomycin. Transient transfections of all cells with miRCURY LNA anti-miR-141, anti-miR-200a and antisense control B (Exiqon, Vedbaek, Denmark; Product No: 4100001-4104908-001) were carried out using Lipofectamine 2000 (Invitrogen, Carlsbad, CA, USA). The miRNA inhibitors used were labeled with fluorescein (6-FAM) (Exiqon, Vedbaek, Denmark). The initial concentrations of both anti-miRs and control B were 50 µM. Cells were seeded in 24-well plates 24 h before transfection at a density of 40,000 cells/well and cultured at 37 °C in a 5% CO_2_-humidified incubator. Transfection efficiency was assessed under the fluorescence microscope using phase contrast (PC) and fluorescein isothiocyanate (FITC) filter sets (Nikon, Tokyo, Japan).

### 2.6. Relative Expression of DLC-1 and ZEB2

We quantified the expression of *DLC-1* and *ZEB2* predicted to be targeted by miR-141 and miR-200a, respectively, using quantitative real-time polymerase chain reaction. Total RNA from the transfected cells were isolated at 24, 48 and 72 h using miRNeasy Mini Kit (Qiagen, Valencia, CA, USA). Up to 2 µg of total RNA was reverse-transcribed to cDNA in a final reaction of 20µl using the High-Capacity RNA to cDNA Kit (Applied Biosystem, Foster City, CA, USA; Cat No: 4387406) following the manufacturer’s instructions. Gene expression was quantified using Taqman Pre-Designed Gene Expression Assays (Hs00183436_m1 for *DLC-1* with NCBI reference: NM_001164271 and Hs002076091_m1 for ZEB2 with NCBI reference: NM_001171653.1) together with Taqman Fast Advanced Master Mix (Applied Biosystem, Foster City, CA, USA; Cat No: 4444964) in accordance to protocols. Relative expression was calculated using the comparative method with Hs02758991_g1 for *GAPDH* with NCBI reference: NM_001256799 as the calibrator. Experiments were done in duplicate. Statistical analysis was performed using a student’s *t*-test, and *p* < 0.05 was considered statistically significant.

### 2.7. Cell Viability, Migration and Invasion Assays

Cell viability, migration and invasion were assayed using PrestoBlue cell viability reagent (Thermo Fisher Scientific, Waltham, MA, USA), QCM 24-well Fluorometric Cell Migration Assay (Millipore, Billerica, MA, USA) and QCM 24-well Cell Invasion Assay (Millipore, Billerica, MA, USA), respectively. The assays were performed at 24, 48 and 72 h post-transfection. Transfected cells stained with PrestoBlue reagent were incubated for 30 min at 37 °C in 5% CO_2_-humidified conditions. For migration and invasion assays, 300 μL of cell suspension (containing 0.5–1.0 × 10^6^ cells/mL in chemo-attractant-free media) was added to the upper chambers (24-well inserts with 8-µm pore size). The lower chambers were filled with 500 µL of media supplemented with 10% fetal bovine serum. Cells in the upper chambers were removed by pipetting out the remaining cell suspension after 24 h of incubation at 37 °C in a 5% CO_2_-humidified incubator. Migrated and invaded cells were dislodged completely from the underside of the inserts using the cell detachment solution and stained with lysis buffer/CyQuant GR dye solution. Fluorescence measurement for all the assays was performed on the Varioskan Flash ELISA reader (Thermo Fisher Scientific, Waltham, MA, USA). Functional assays were performed in duplicate. Statistical analysis was performed using a student’s *t*-test, and *p* < 0.05 was considered statistically significant.

## 3. Results

### 3.1. Differentially Expressed microRNAs in Metastatic SOC Compared to Normal

The NormFinder identified miR-27a with the lowest standard deviation, and this was chosen as the endogenous miRNA control to be normalized with all the samples. A total of 48 miRNAs were differentially expressed significantly in metastatic SOC compared to normal (*p* < 0.05), in which 22 and 26 miRNAs were up- and downregulated, respectively. The most significantly upregulated miRNAs with log_2_-fold changes >2 were miR-106a, miR-141, miR-182, miR-183, miR-18a, miR-19a, miR-200a/b/c, miR-205, miR-203, miR-20b, miR-21, miR-31 and miR-7. MiR-133a, miR-145, miR-195, miR-202, miR-1 and miR-100 were among the most downregulated miRNAs with log_2_-fold changes <−2. The complete list of significantly dysregulated miRNAs is presented in Table 2.

**Figure 1:**
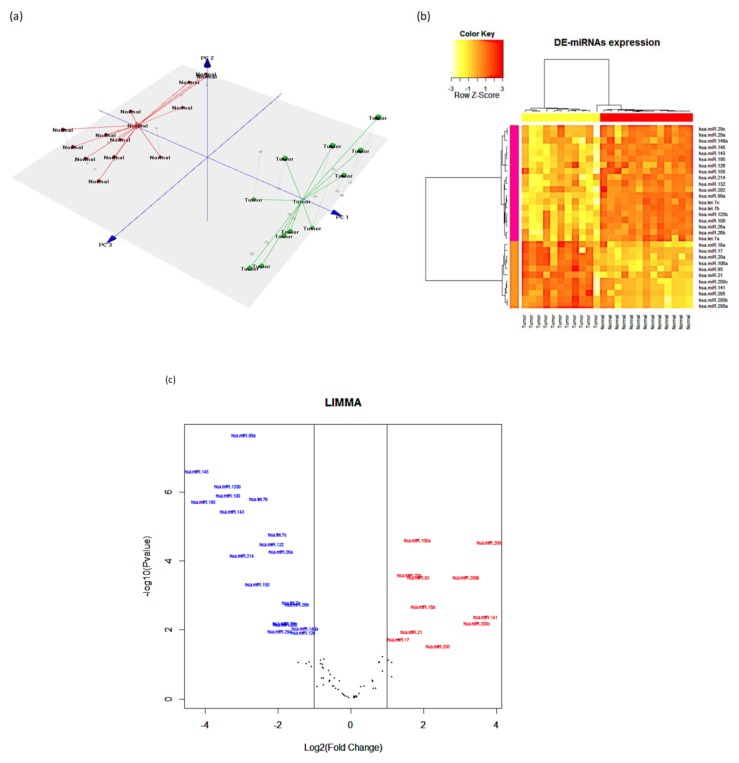
MicroRNA (MiRNA) expression profiling in metastatic serous ovarian cancer (SOC) compared to normal tissues. (**a**) Principal component analysis (PCA) shows the grouping of samples into cancer group (green lines) and normal (red lines). (**b**) Heatmap demonstrating the differentially expressed miRNAs in the tissue samples. Red shows upregulated miRNAs, and yellow shows downregulated miRNAs. (**c**) Volcano plot showing the relationship between the −log_10_ *p*-value and the log_2_-fold change in metastatic SOC compared to normal.

The differentially expressed miRNAs in metastatic SOC compared to normal samples are represented by the principal component analysis (PCA) and the heatmap analysis, as shown in Figure 1a and Figure 1b, respectively.

**Figure 2:**
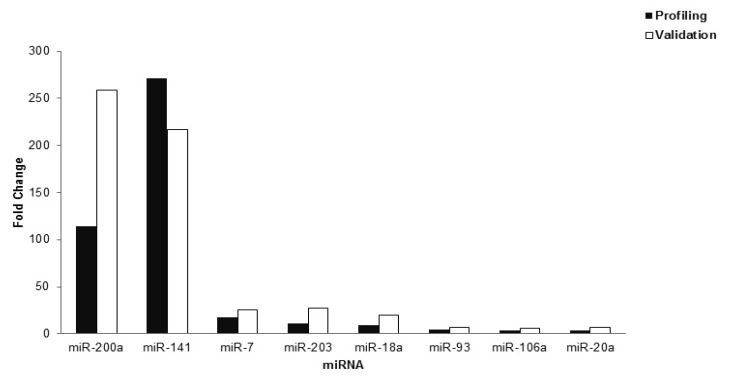
Validation of eight upregulated miRNAs found in the profiling study. The validation data shows good concordance with the profiling data (*p* < 0.05), thus confirming the profiling results.

The PCA and heatmap diagram clearly distinguished the samples into two different groups: metastatic SOC and normal samples. The volcano plot in Figure 1c shows the relationship between the −log_10_ *p*-value and the log_2_-fold change in metastatic SOC compared to normal samples. Validation of the eight upregulated miRNAs confirmed the profiling results, as shown in Figure 2.

### 3.2. Predicted Targeted Genes for miR-141 and miR-200a

**Figure 3:**
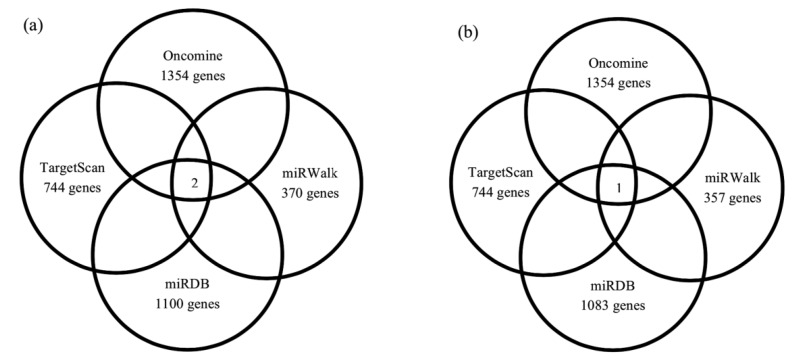
Venn diagrams showing the overlapping targeted genes for selected miRNAs as predicted by the bioinformatic analysis. (**a**) Two overlapping targeted genes for miR-141 were identified, namely *DLC-1* and *ZEB2*. (**b**) MiR-200a is a single predicted miRNA to target the *ZEB2* gene, which overlaps in all datasets.

Oncomine search discovered the dataset from Adib et al. that listed a total of 1354 genes (*p* < 0.05) upregulated in SOC compared to normal. Analysis with TargetScan showed 744 predicted targeted genes for miR-141, 370 genes with miRWalk and 1100 genes with miRDB. MiR-200a was found to target 744 genes predicted by TargetScan, 357 genes by miRWalk and 1083 genes by miRDB. As shown in the Genn-Venn diagram (Figure 3), overlapping the datasets from the Oncomine and miRNA target prediction tools revealed ZEB2 and Deleted in Liver Cancer 1 (DLC-1) as the targeted genes for miR-141 and *ZEB2* as the targeted gene for miR-200a.

### 3.3. The Expression of DLC-1 and ZEB2 Increased Following miR-141 and miR-200a Inhibition, Respectively

**Figure 4:**
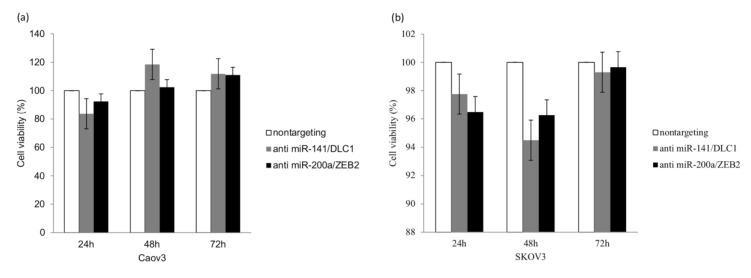
Relative expression of *DLC-1* and *ZEB2* increased following treatment with miRNA inhibitors. (**a**) In Caov3 cells, the relative expression of *DLC-1* and *ZEB2* increased after 72 h of transfection with anti-miR-141 and anti-miR-200a, respectively. (**b**) Similarly, miR-141 and miR-200a inhibition in SKOV3 cells led to increased *DLC-1* and *ZEB2* expression, respectively, after 72 h. Statistical analysis was performed using a student’s *t*-test. The graph represents the mean ± standard error of duplicate experiments.

**Figure A1:**
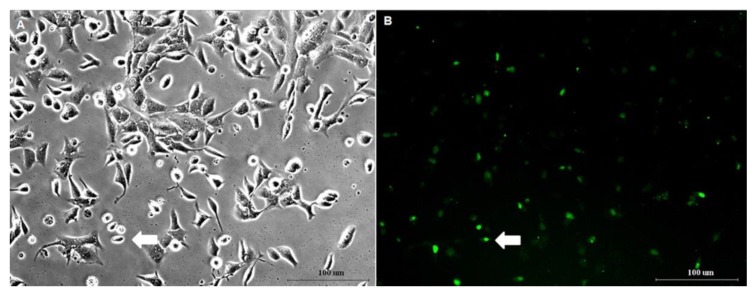
(**A**) SKOV-3 cells under fluorescence microscope (phase contrast), x200. (**B**) SKOV-3 cells under fluorescence microscope (FITC), x200. Green fluorecein (arrow) indicates miR-141 inhibitor successfully transfected into the cell. Transfection efficiency was between 60–70%.

Transfection efficiency was 60% to 70%, as observed under the fluorescence microscope using a FITC filter set (Figure A1). As shown in Figure 4, the relative expression of *DLC-1* and *ZEB2* in Caov3 cells increased by 1.5- and 1.4-fold after 72 h of transfection with anti-miR-141 and anti-miR-200a, respectively. MiR-141 and miR-200a inhibition in SKOV3 cells caused the relative expression of *DLC-1* and *ZEB2* to rise by 1.2- and 1.5-fold, respectively, after 72 h of transfection (Figure 4). However, the changes observed were not statistically significant.

### 3.4. MicroRNA Inhibition Reduced Cell Viability while Enhancing Cell Migration and Invasion

**Figure 5:**
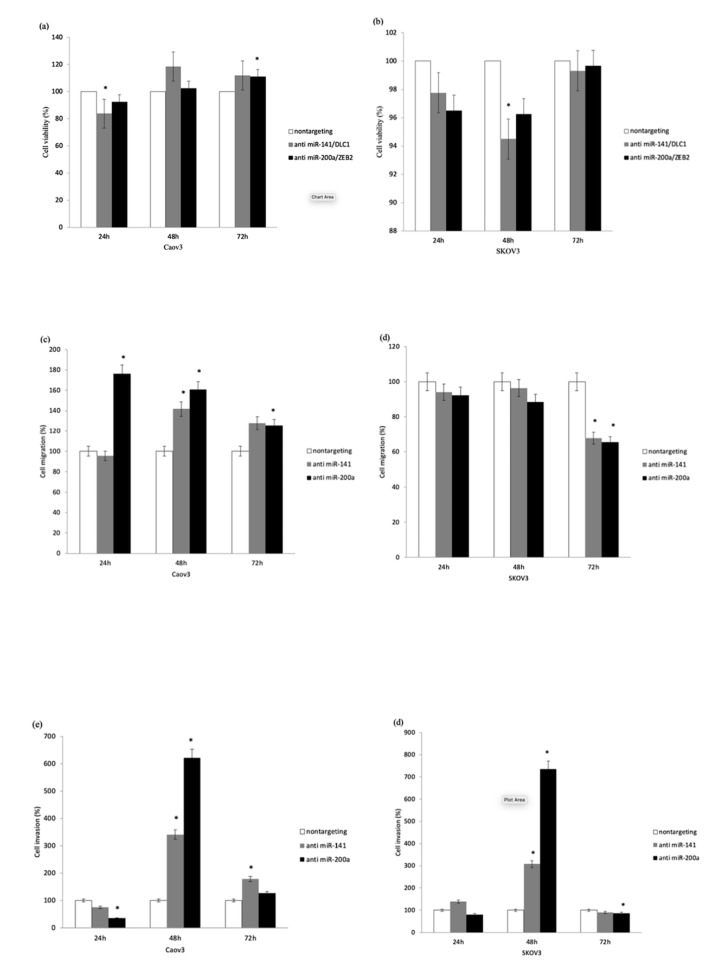
Functional assays performed on Caov3 and SKOV3 cells. (**a**) MiR-141 inhibition in Caov3 and (**b**) SKOV3 cells caused a significant reduction in cell viability after 24 h and 48 h, respectively. Anti-miR-200a treatment did not cause a reduction in cell viability in both cell lines. (**c**) In Caov3 cells, treatment with anti-miR-141 caused enhanced cell migration significantly after 48 h, while anti-miR-200a treatment led to the highest increase in cell migration after 24 h. (**d**) Increased cell migration, however, was not observed in SKOV3 cells treated with miRNA inhibitors. (**e**,**f**) MiR-141 and miR-200a inhibition in both cell lines led to the highest significant increase in cell invasion after 48 h. Statistical analysis was performed using a student’s *t*-test. The graph represents the mean ± standard error of duplicate experiments. * *p* < 0.05.

As shown in Figure 5a, the percentage of viable Caov3 cells reduced significantly to 84% after 24 h of transfection with anti-miR-141 (*p* < 0.05). Significant reduction in cell viability (94.5%) was also observed in SKOV3 cells after 48 h transfection with anti-miR-141 (*p* < 0.05). No significant decrease in cell viability was observed in both cells treated with anti-miR-200a, as shown in Figure 5a,b.

Transfection with anti-miR-141 in Caov3 cells increased the percentage of migrated cells significantly to 142% after 48 h (Figure 5c), while miR-200a inhibition caused the largest increase in cell migration (176%) after 24 h (*p* < 0.05), as shown in Figure 5d. However, miR-141 and miR-200a suppression did not appear to increase the percentage of migrated cells in SKOV3 cells, as shown in Figure 5d.

As depicted in Figure 5e, inhibition of miR-141 and miR-200a led to a significant increase in the percentage of invaded cells in Caov3 and SKOV3 cells with the largest rise seen at 48 h (*p* < 0.05). MiR-200a inhibition seemed to have the greatest effect on cell invasion, with a 621% and 735% upsurge of invading cells observed in Caov3 (Figure 5e) and SKOV3 cells (Figure 5f), respectively.

## 4. Discussion

This study was initiated with the hope of identifying histotype-specific miRNA expression patterns with a greater emphasis on cancers with metastatic potential. Many profiling studies have been done on ovarian cancer across different continents and populations, such as the United States of America, China, Korea and Taiwan [9,19,20,21]. In this paper, the differentially expressed miRNAs have been successfully categorized into two main clusters (up- and downregulated miRNAs) for the Malaysian population. Another Malaysian study has demonstrated the differentially expressed miRNAs in formalin-fixed paraffin-embedded (FFPE) ovarian tissue samples [10]. Our study complements the current literature by showing the aberrantly expressed miRNAs in fresh ovarian tissue samples.

A large number of the upregulated miRNAs determined from our profiling experiment were consistent with those which have been reported, namely miR-106a [19], miR-141 [10,19,22], miR-15a [20,22], miR-17 [19], miR-182 [10,16,19], miR-18a [10,22], miR-200a [10,19,22], miR-200b, miR-200c [10,19,22], miR-203 [10,22], mir-205 [10], miR-20a [22], miR-20b [10], miR-7 [10] and miR-93 [10]. However, the expression of miR-106b and miR-183 were inconsistent with the study by Dahiya et al. in which these miRNAs were found to be downregulated in epithelial ovarian cancer compared to normal [23]. MiR-21, miR-210 and miR-31 were found to have different expression in different studies [10,20,22]. The discrepancies in miRNA expression observed from these studies may be contributed by the different types of ovarian tissue samples used (fresh samples, FFPE and cells); histological subtypes of epithelial ovarian cancer included in the study (serous, endometrioid, mucin and clear cell); the profiling platforms used (qPCR, microarray and deep sequencing) and the normal tissues used as controls (whole normal ovary and normal ovarian surface epithelial cells).

Nevertheless, despite the many differences in the methods employed in different studies, good concordance of the overlapping upregulated miRNAs was observed, as shown in the list above. Potentially effective biomarkers for diagnostic, treatment or prognostic purposes may be unearthed from this unique overlapping miRNA list, thus providing useful insights for the ongoing translational research, from biomarker discoveries in the lab to clinical practices in the healthcare setting. This study is also the first to report on the upregulation of miR-19a in metastatic SOC compared to normal, making it a novel discovery. This new finding will certainly add to the existing knowledge of the upregulated miRNAs in metastatic SOC compared to normal.

In the list of upregulated miRNAs as determined from our profiling study, miR-141 was found to have the highest expression in metastatic SOC compared to normal, with log2-fold changes more than 9 (Table 2). MiR-141 is a member of the miR-200 family, alongside other members, namely miR-200a/b/c and miR-429 [24]. In this paper, miR-200a/b/c were also shown to have high expression in metastatic SOC compared to normal, with log2-fold changes more than 7. The high expression of the miR-200 family in metastatic SOC compared to normal was also observed in previous profiling studies [10,19,22]. The high expression of the miR-200 family found in our study has led us to choose miR-141 and miR-200a to be further experimented (in vitro) to determine their interactions with the targeted genes and their roles in ovarian cancer growth and progression.

The bioinformatics analysis conducted in this study has shown that *DLC-1* and *ZEB2* were the predicted targeted genes for miR-141 and miR-200a, respectively, as shown in Figure 3. The gene *DLC-1* is a tumor suppressor, and *ZEB2* is a transcriptional repressor, which have been shown to be downregulated in ovarian cancers compared to normal tissues [25]. We hypothesized that miR-141 and miR-200a inhibition in ovarian cancer cells will increase the expression levels of their targeted genes. Our results demonstrated increased *DLC-1* and *ZEB2* expression following miR-141 and miR-200a inhibition, respectively (Figure 4). However, the changes in fold change observed were not statistically significant, and some may argue that the changes seemed minimal to exert substantial effects on cancer cell regulation (1.2- to 1.5-fold changes). We recognized these limitations; nevertheless, the findings in this paper may provide preliminary insights on the interaction between miR-141 and miR-200a and *DLC-1* and *ZEB2*, respectively, in SOC. A study by Bendroaite et al. confirmed that *ZEB2* is the targeted gene for the miR-200 family in ovarian cancer using the luciferase-*ZEB2* 3′-UTR reporter constructs and miR-200 family expression plasmids [25]. The regulation of *DLC-1* by miR-141 has also been shown in colorectal cancer [26]. Our study is the first to demonstrate the possible regulation of *DLC1* by miR-141 in SOC; thus, experiments using the luciferase reporter assay can be performed in the future to confirm *DLC-1* as the targeted gene for miR-141. DLC-1 acts as a tumor-suppressor gene, and its low expression was associated with poor prognosis in lung cancer [27]. A meta-analysis study involving 1815 cancer cases demonstrating a low expression of DLC-1 was associated with advanced stages of cancer, including ovarian cancer [28].

The overexpression of miR-141 in ovarian cancer cell lines was shown to cause increased resistance to cisplatin, thus enhancing cancer growth [29]. MiR-141 and miR-200a were also found to have effects on ovarian tumor growth through the oxidative stress response control mechanism. Increased expression of these miRNAs has been shown to increase tumor growth in mouse models [11]. We have shown that cell viability was significantly reduced in ovarian cancer cells following transfection with antimiR-141, as shown in Figure 5a. In view of our findings, we proposed that treatment with antimiR-141 may result in an antitumorigenic effect via a reduction in cell viability. However, significant reduction in cell viability was only seen in cells treated with anti-miR-141 but not with anti-miR-200a. This may be due to the different molecular pathways or cell signals that promote ovarian cancer growth in relation to these miRNAs, such as the P13K pathway and the estrogen receptor pathway [30].

In this study, we observed that transfection with miRNA inhibitors led to a significant rise in ovarian cancer cell migration and invasion, as shown in Figure 5c,d and Figure 5e,f. Cancer progression was largely attributed to the epithelial-to-mesenchymal transition (EMT), which was associated with tumor migration, invasion, metastasis and treatment resistance [31]. EMT is characterized by the loss of E-cadherin, an important adhesion molecule of epithelial cells, resulting from the overexpression of transcriptional repressors *ZEB1* or *ZEB2*, Snail 1/2 and TWIST [32,33]. Suppression of E-cadherin expression leads to the acquisition of mesenchymal and migratory characteristics and metastasis [34]. In ovarian cancer, members of the miR-200 family have been implicated with EMT in which *ZEB1* and *ZEB2* were their targets [25]. Upregulation of the miR-200 family is thought to impede EMT and suppress metastasis by augmenting E-cadherin expression through the direct targeting of *ZEB1* and *ZEB2*. Overexpression of the miR-200 family was also found to inhibit cell mobility significantly in ovarian cancer cells [12]. Conversely, the miR-200 family repression leads to reduced E-cadherin expression and loss of cell-cell adhesion, thus enhancing cell invasion and migration [35]. This understanding is further substantiated by the results of our cell migration and invasion experiments.

Nevertheless, it is notable that treatment with miRNA inhibitors led to enhanced cell migration in Caov3 cells, but migration was significantly reduced by almost 30% in SKOV3 cells. This discrepancy may be attributed to the sources of the cell lines used. The cell line Caov3 was derived from primary epithelial ovarian adenocarcinoma, while SKOV3 was established from the ascites of epithelial ovarian adenocarcinoma (American Type Culture Collection, Manassas, VA, USA). Moreover, the putative histological subtypes of the two cell lines used may also contribute to the discrepancy. Both Caov3 and SKOV3 cell lines have TP53 mutations, but SKOV3 cells also have ARID1A mutation, making Caov3 putative histology as serous, while SKOV3 very much represents the endometrioid histological subtype [36]. As such, cell lines with molecular properties which best represent the histological subtype under study would be an important aspect to be addressed in future studies.

Although cell migration was seen to increase only in Caov3 cells, cell invasion was observed to rise significantly in both Caov3 and SKOV3 cells following transfection with miRNA inhibitors, as shown in Figure 5e,f. This may be due to the differences in the molecular process of cell migration and invasion, in which cancer cells can only invade if migration occurs but migration may occur without invasion [37]. Migration refers to the movement of cancer cells from one site to another site, but invasion involves the rupture of the cell membrane with the aid of the protein matrix metalloproteases [37].

## 5. Conclusions

In conclusion, our study has demonstrated the differentially expressed miRNAs in metastatic SOC compared to normal, with miR-19a identified as a novel miRNA to be upregulated. Possible regulation of *DLC-1* by miR-141 in SOC was shown, and this finding may serve as an impetus for future experiments to be done more extensively. The functional roles of miR-141 and miR-200a add to the existing knowledge of how these miRNAs contribute to SOC carcinogenesis. This report contributes to the understanding of miRNA expression profiling in metastatic SOC and the molecular aspects underlying its growth and progression.

## Figures and Tables

**Table 1:** The patient information of the ovarian tissue samples used in the study.

Characteristics	Cancer Samples (N (%))	Normal
Number of samples	11	13
Mean age ± S.D. (year)	49.9 ± 10.6	51.9 ± 6.7
Race		
Malay	11 (100%)	5 (38.5%)
Chinese	0	6 (46.2%)
Indian	0	2 (15.4%)
Stage		
III	9 (81.8%)	
IV	2 (18.2%)	
Low-grade	1 (9.1%)	
High-grade	10 (90.9%)	

**Table 2:** The list of differentially expressed miRNAs in metastatic serous ovarian cancer (SOC) compared to normal.

miRNAs	Kruskal-Wallis (Adjusted *p* Value)	Limma (Adjusted *p* Value)	Log_2_-Fold Change
Upregulated			
miR-141	0.000163	3.33 × 10^−14^	9.419905
miR-200c	0.000163	3.51 × 10^−11^	8.867292
miR-200b	0.000163	2.67 × 10^−11^	7.872327
miR-205	0.000163	9.32 × 10^−11^	7.804692
miR-200a	0.000163	3.33 × 10^−14^	7.694753
miR-182	0.000163	1.99 × 10^−12^	6.125206
miR-183	0.000163	1.21 × 10^−10^	4.882529
miR-7	0.000211	2.60 × 10^−6^	3.953067
miR-203	0.000163	2.60 × 10^−6^	3.227105
miR-18a	0.000241	4.79 × 10^−7^	3.065583
miR-31	0.027471	0.003668	2.604579
miR-20b	0.000281	2.02 × 10^−5^	2.461332
miR-21	0.000777	2.02 × 10^−5^	2.115933
miR-19a	0.007898	0.009027	2.069257
miR-106a	0.000163	5.70 × 10^−10^	2.032367
miR-20a	0.000163	7.64 × 10^−8^	1.914742
miR-93	0.000186	2.70 × 10^−6^	1.849742
miR-17	0.003409	8.56 × 10^−5^	1.668148
miR-106b	0.000281	1.41 × 10^−5^	1.337312
miR-210	0.013004	0.012497	1.202425
miR-30d	0.005935	0.005966	1.202272
miR-15a	0.001138	0.000167	1.197055
Downregulated			
miR-202	0.000163	1.53 × 10^−8^	−5.74109
miR-133a	0.000163	7.77 × 10^−9^	−4.25237
miR-145	0.000163	4.25 × 10^−8^	−4.24071
miR-195	0.000163	7.13 × 10^−7^	−4.04207
miR-125b	0.000163	1.47 × 10^−11^	−3.82333
miR-100	0.000163	1.09 × 10^−10^	−3.67522
miR-1	0.000163	1.13 × 10^−7^	−3.47619
miR-143	0.000163	2.50 × 10^−7^	−3.41547
miR-214	0.000541	2.97 × 10^−5^	−3.39572
miR-99a	0.000163	1.58 × 10^−10^	−2.79158
miR-9	0.000186	1.89 × 10^−8^	−2.73715
miR-150	0.000241	3.79 × 10^−6^	−2.55192
let-7b	0.000241	2.04 × 10^−7^	−2.53452
miR-34a	0.007898	0.000167	−2.17351
miR-132	0.000777	7.67 × 10^−6^	−2.1695
miR-29a	0.000211	7.96 × 10^−7^	−2.13643
miR-26a	0.000777	1.03 × 10^−5^	−1.91756
let-7c	0.000211	4.70 × 10^−7^	−1.84412
miR-10b	0.040545	0.00972	−1.77229
miR-29c	0.002916	0.000189	−1.69908
let-7a	0.003409	0.000534	−1.63707
miR-126	0.001138	0.000189	−1.57362
miR-26b	0.002517	0.000534	−1.47826
miR-423-5p	0.000541	3.96 × 10^−5^	−1.41021
miR-148a	0.007028	0.003807	−1.25192
miR-29b	0.017849	0.007969	−1.07275
